# An Evidence-Based Approach for Choosing Post-exercise Recovery Techniques to Reduce Markers of Muscle Damage, Soreness, Fatigue, and Inflammation: A Systematic Review With Meta-Analysis

**DOI:** 10.3389/fphys.2018.00403

**Published:** 2018-04-26

**Authors:** Olivier Dupuy, Wafa Douzi, Dimitri Theurot, Laurent Bosquet, Benoit Dugué

**Affiliations:** Laboratoire MOVE (EA6314), Faculty of Sport Sciences, University of Poitiers, Poitiers, France

**Keywords:** meta-analysis, recovery, DOMS, fatigue, inflammation, muscle damage, intervention efficiency

## Abstract

**Introduction:** The aim of the present work was to perform a meta-analysis evaluating the impact of recovery techniques on delayed onset muscle soreness (DOMS), perceived fatigue, muscle damage, and inflammatory markers after physical exercise.

**Method:** Three databases including *PubMed, Embase*, and *Web-of-Science* were searched using the following terms: (“recovery” or “active recovery” or “cooling” or “massage” or “compression garment” or “electrostimulation” or “stretching” or “immersion” or “cryotherapy”) and (“DOMS” or “perceived fatigue” or “CK” or “CRP” or “IL-6”) and (“after exercise” or “post-exercise”) for randomized controlled trials, crossover trials, and repeated-measure studies. Overall, 99 studies were included.

**Results:** Active recovery, massage, compression garments, immersion, contrast water therapy, and cryotherapy induced a small to large decrease (−2.26 < g < −0.40) in the magnitude of DOMS, while there was no change for the other methods. Massage was found to be the most powerful technique for recovering from DOMS and fatigue. In terms of muscle damage and inflammatory markers, we observed an overall moderate decrease in creatine kinase [SMD (95% CI) = −0.37 (−0.58 to −0.16), I^2^ = 40.15%] and overall small decreases in interleukin-6 [SMD (95% CI) = −0.36 (−0.60 to −0.12), I^2^ = 0%] and C-reactive protein [SMD (95% CI) = −0.38 (−0.59 to−0.14), I^2^ = 39%]. The most powerful techniques for reducing inflammation were massage and cold exposure.

**Conclusion**: Massage seems to be the most effective method for reducing DOMS and perceived fatigue. Perceived fatigue can be effectively managed using compression techniques, such as compression garments, massage, or water immersion.

## Introduction

Scientific evidence gathered during the past few decades has allowed the identification of the most efficient training strategies for improving physical performance. However, maximizing the performance capacity of an athlete is not only a matter of training. It also depends on an optimal balance between training and recovery in order to prevent maladaptation to accumulated psychological and physiological stresses induced by the training load (Meeusen et al., 2013; Soligard et al., 2016).

Indeed, competition and training can induce repeated eccentric contractions (Ispirlidis et al., 2008) and tissue vibrations (Friesenbichler et al., 2011) that can lead to muscle damage (i.e., disruption of structural proteins in muscle fibers and/or connective tissues), subsequent tissue inflammation, delayed onset muscle soreness (DOMS), and increased perceived fatigue (Cheung et al., 2003; Dugué, 2015). Changes in the blood concentrations of muscle damage indicators [i.e., creatine kinase (CK)] and inflammatory biomarkers [C-reactive protein (CRP) and interleukin-6 (IL-6)] that are observed after exercise and are associated with the occurrence of DOMS can also be used to achieve skeletal muscle recovery (Bishop et al., 2008; Banfi et al., 2009; Stacey et al., 2010; Leal Junior et al., 2011).

These exercise-induced perturbations can lead to a temporary reduction in muscular force (Brown et al., 1997; Goodall and Howatson, 2008; Mackey et al., 2008), a disturbed sense of joint position (Saxton et al., 1995; Paschalis et al., 2007), decreased physical performance (Twist and Eston, 2009; Burt and Twist, 2011), and/or an increased risk of injury (Cheung et al., 2003; Barnett, 2006). In this context, it is important for coaches and sports scientists to optimize the recovery period in order to manage muscle damage and alleviate DOMS, inflammation, and fatigue, thereby allowing the athlete to feel less fatigued and decrease the risk of injury (Cheung et al., 2003; Soligard et al., 2016) or maladaptation to the training load (Kenttä and Hassmén, 1998; Meeusen et al., 2013).

In terms of the training frequency of elite athletes, a brief duration between two training sessions may not be enough to achieve a complete recovery (Barnett, 2006; Minett and Duffield, 2014), which is defined as the return to homeostasis of various physiological systems following metabolic and inflammatory challenges and muscle damage induced by exercise training sessions (Hausswirth and Le Meur, 2011). The interaction between the training load, subsequent fatigue and adaptation is complex (Fröhlich et al., 2014; Halson et al., 2014; Roberts et al., 2015) and may be modulated (positively or negatively) by the recovery strategy (Minett and Costello, 2015). Therefore, the choice of recovery techniques is of utmost importance to enable the athlete to perform during the next training session feeling rested, not fatigued, healthy, and injury-free (Hausswirth and Le Meur, 2011).

Several kinds of recovery interventions have been proposed to improve recovery after physical exercise (Barnett, 2006; Bishop et al., 2008; Robson-Ansley et al., 2009; Nédélec et al., 2013; Kovacs and Baker, 2014), including the following: compressive techniques, such as massage (Kargarfard et al., 2016), compressive garments (Marqués-Jiménez et al., 2016), or water immersion (Leeder et al., 2012); electrostimulation (Bieuzen et al., 2014a); stretching (Herbert and Gabriel, 2002); anti-inflammatory interventions relying on cold exposure, such as cryotherapy (Costello et al., 2015) or cold water immersion (Poppendieck et al., 2013); and active recovery (Andersson et al., 2008). Depending on the timing and the context, these interventions can target either central or peripheral mechanisms (Minett and Duffield, 2014). The mechanisms underlying the beneficial effects of recovery may be technique dependent. However, most of them are share common processes that allow decreases in exercise-induced muscle damage and inflammation. Such mechanisms enable a reduction in the space available for swelling and oedema formation, thereby limiting fluid diffusion into the interstitial space and facilitating the transport of metabolites/neutrophils/damage proteins from the muscle to the blood through changes in blood and lymph flow (Barnett, 2006; Bishop et al., 2008; Robson-Ansley et al., 2009; Nédélec et al., 2013; Kovacs and Baker, 2014).

Our knowledge of the dose-response relationship between different protocols and the magnitude of their impact on DOMS, fatigue, muscle damage, and inflammatory markers has greatly improved for each of the recovery techniques, and some evidence-based recommendations are now available. The difficulty for athletes, coaches, sports scientists, and medical staff is that very few studies have compared the impact of these techniques with the others, and there is a lack of information to facilitate choosing the best adequate recovery techniques. Furthermore, no comparisons of the impacts of different recovery techniques have been presented using a meta-analysis thus far. Therefore, the aim of the present work is to compare the effects of the most commonly used recovery techniques on muscle damage, DOMS, inflammation, and the perception of fatigue induced by physical exercise through a meta-analysis of the scientific literature.

## Methods

### Literature search

**Figure 1:**
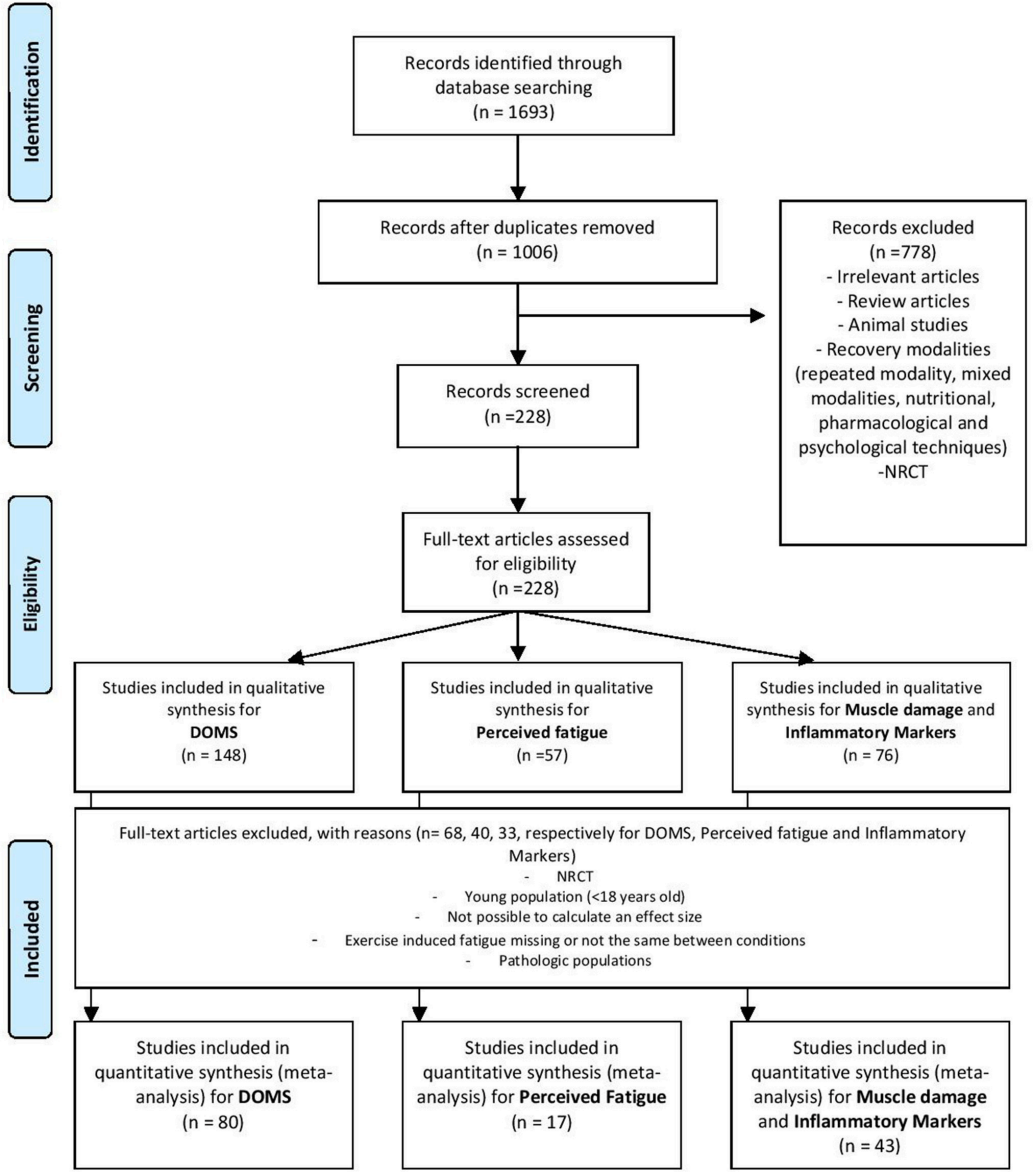
Flow chart.

Three databases, namely, *PubMed* (1949 to 2017), *Embase* (1974 to 2017), and *Web-of-Science* (1945 to 2017), were searched using the following terms: (“recovery” or “active recovery” or “cooling” or “massage” or “compression garment” or “electrostimulation” or “stretching” or “immersion” or “cryotherapy”) and (“delayed onset muscle soreness (DOMS)” or “perceived fatigue” or “creatine kinase (CK)” or “C-reactive protein (CRP)” or “interleukin-6 (IL-6)”) and (“after exercise” or post-exercise”) for “English” or “French-language” randomized controlled trials, crossover trials, and repeated-measure studies. In addition, we completed our literature search with two databases that contain theses and dissertations (*Kinpubs* and *Sport-Discus*) and thereby included “gray literature” (i.e., literature that is difficult to locate or retrieve, Moher et al., 1999). The reference lists of the articles obtained were searched manually to obtain additional studies that were not electronically identified. This led to the identification of 1693 potential studies for inclusion in the analysis (Figure 1). The last update was made on November 6, 2017.

### Selection criteria

Studies were eligible for inclusion if the met the following criteria: (1) they implemented an exercise intervention followed by a recovery intervention and provided relevant details about the procedures, including the modality, duration and intensity of exercise; (2) they detailed the modality and timing of the recovery intervention [repeated sessions of recovery and/or combined modalities were not included (e.g., ice + massage)]; (3) the outcome included valid tests and measures of DOMS, perceived fatigue, muscle damage, and inflammation markers in healthy humans adults (>18 years to <65 years); and (4) they reported the number of participants and all the necessary data to calculate effect sizes. Studies were excluded if they presented results from a previous publication (duplicated data). Our study protocol followed the Quality of Reporting of Meta-analyses (QUOROM) (Moher et al., 1999) and the Preferred Reporting Items for Systematic Reviews and Meta-Analyses (PRISMA) statements (Moher et al., 2009). The exclusion criteria are detailed in Figure 1.

### Coding for the studies

Two independent reviewers who were blinded to authors, affiliations, and the publishing journal independently read and coded all the included articles (Jadad et al., 1996) using the PEDro scale (Maher et al., 2003) and the van Tulder scale (van Tulder et al., 2003). Following this quality assessment, the same reviewers read and coded each of the studies and assessed the following moderators: training status (competitive athletes, recreational athletes, or inactive people), sex (male, female, or both), exercise duration (minutes), exercise type [cardiorespiratory exercise (continuous or intermittent) or resistance exercise], exercise intensity (very light, light, moderate, vigorous, or maximal; according to the criteria of Garber et al., 2011), type of recovery (active recovery, stretching, massage, compression garment, electrostimulation, immersion, contrast water therapy, cryotherapy, or hyperbaric therapy), temperature of the immersion (<15°C, 15 to 35°C, or >35°C), level of immersion (arms, legs, or whole body), temperature of the cryotherapy chamber (>−80°C, −80 to −110°C, or <−110), and timing of the measurements (<6, 24, 48, 72, >96 h). An interval scale was used for the coding of exercise duration, while a nominal scale was used for the coding of the other moderators. Any disagreement between both reviewers was discussed in a consensus meeting, and unresolved items were addressed by a third reviewer for resolution.

### Statistical analysis

Standardized mean differences (SMDs) were calculated for each experimental group using Hedges' g (Hedges, 1981). In the studies that used multiple measures of a parameter of interest, a single composite SMD was calculated to estimate the overall effect (Borenstein et al., 2009). We decided *a priori* to use a random-effects model because the effect of a recovery intervention might differ according to the type of fatiguing exercise, training status, sex, or other moderators. Standardized mean differences were weighted by the inverse of the variance to calculate the overall effect and 95% confidence interval. Cohen's criteria were used to interpret the magnitude of the SMD: < |0.50|: small; |0.50| to |0.80|: moderate; and >|0.80|: large (Cohen, 1988). Statistical heterogeneity, which refers to the percentage of variability between studies that is due to clinical and methodological heterogeneity rather than sampling error, was assessed by the I^2^ statistic (Borenstein et al., 2009). According to Higgins et al. (2003), I^2^ values of 25, 50, and 75% represent low, medium, and high heterogeneity, respectively. The presence of medium or high heterogeneity may provoke further investigation through a subgroup analysis of moderator variables (sex, exercise, training status, technique of recovery, level of immersion, and water temperature), even if the overall effect is considered non-significant (35). Weighted SMDs and standard errors were calculated for each category within the moderator variables; in addition, 95% confidence intervals were calculated to determine whether a given SMD was different from 0. A Q-test based on analysis of variance was performed to test the null hypothesis that the effect of recovery was similar among the categories of a moderator variable (Borenstein et al., 2009). When the null hypothesis was rejected, pairwise comparisons were performed with a Z test. The statistical significance level was set at *P* = 0.05. All calculations were carried out with a spreadsheet (Excel) and Comprehensive Meta-analysis software (www.meta-analysis.com).

## Results

### Overall results

**Figure 2:**
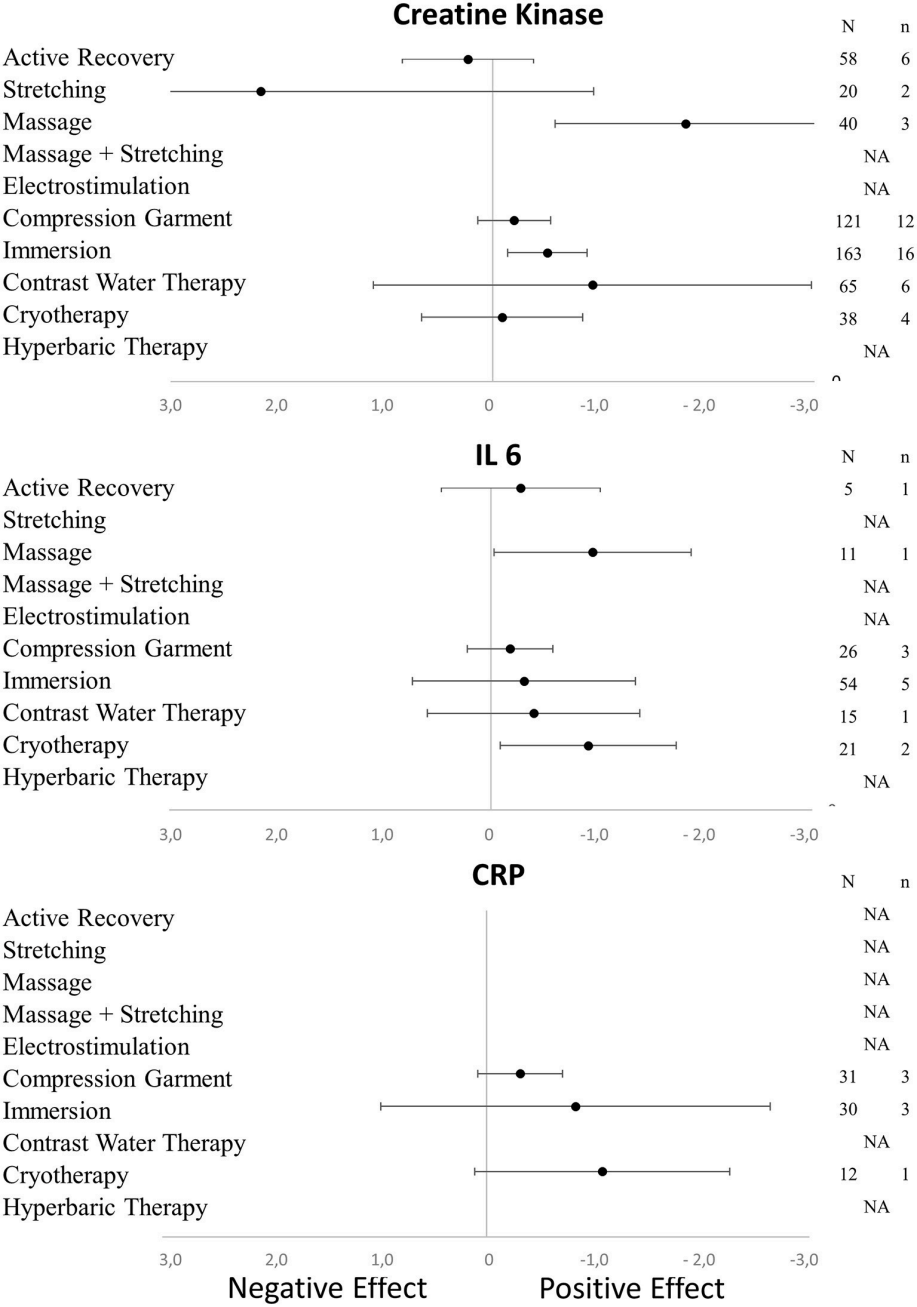
Effects of recovery techniques on the kinetics of muscle damage (CK) and inflammatory markers (CRP and IL-6) (NA, not available; N, number of subjects; n, number of experimental groups).

**Figure 3:**
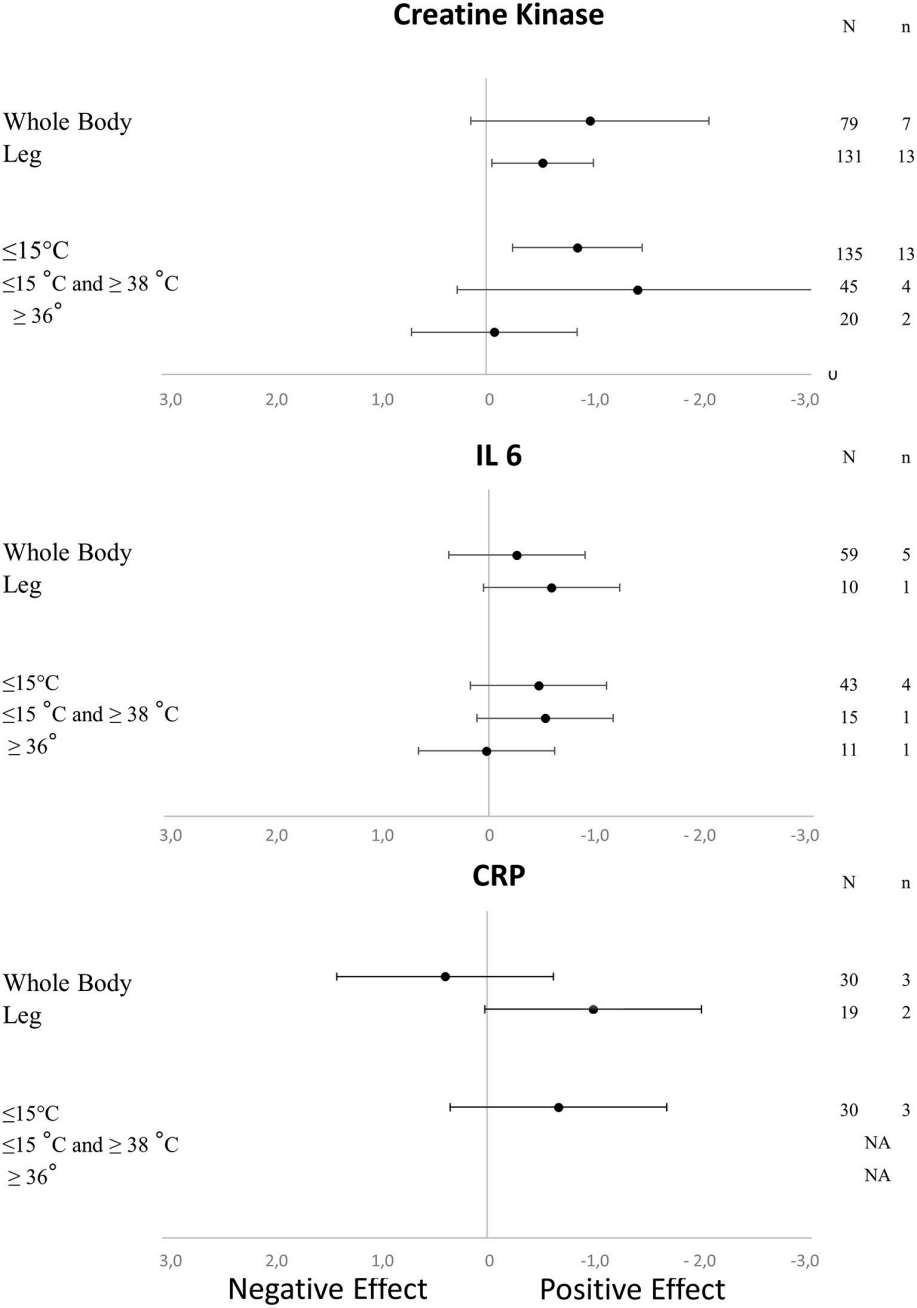
Effects of the characteristics of immersion on the kinetics of muscle damage (CK) and inflammatory markers (CRP and IL-6) (NA, not available; N, number of subjects; n, number of experimental groups).

**Figure 4:**
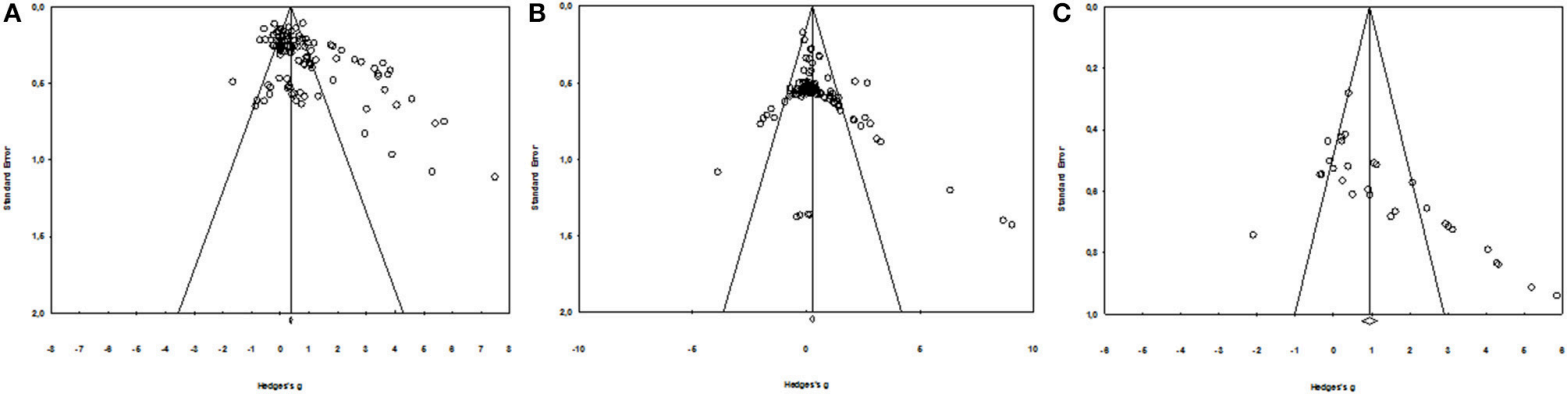
Funnel Plot for DOMS **(A)**, muscle damage, and inflammatory markers **(B)** and perceived fatigue **(C)**.

The literature search identified 1693 potentially relevant publications spanning from 1958 to 2017, of which 99 studies met all the inclusion criteria (80 for DOMS, including 1188 participants and 106 experimental groups; 17 for perceived fatigue, including 266 participants and 27 experimental groups; 19 for inflammatory markers; and 37 for muscle damage marker). The numbers of participants and experimental groups for each inflammatory marker are reported in Figures 2, 3. Funnel plots that were used to identify a possible publication bias are presented in Figure 4. With the exception of DOMS (23 studies out of 57 were distributed toward a positive effect), inflammatory markers (4 studies out of 39 were distributed toward a positive effect), and perceived fatigue (9 studies out of 8 were distributed toward a positive effect), all the other measures were distributed symmetrically about the mean effect size, suggesting that the sampling error was random. We also performed a sensitivity analysis to determine whether the quality score of the studies should be considered as a possible effect modifier. We did not find any difference between groups (the criteria for group allocation was a quality score of 5).

### Effect of recovery methods on DOMS, perceived fatigue, inflammation and muscle damage

The overall SMD indicated a positive effect of a recovery intervention on the magnitude of DOMS [SMD (95% CI) = −0.78 (−0.61 to −0.94), I^2^ = 56.62%] and perceived fatigue [SMD (95% CI) = −1.40 (−0.89 to −1.92), I^2^ = 33%]. The presence of statistical heterogeneity justified the subgroup analysis of moderator variables, including population characteristics (sex and training status), as well as exercise (type, intensity, and duration) and recovery characteristics.

**Figure 5:**
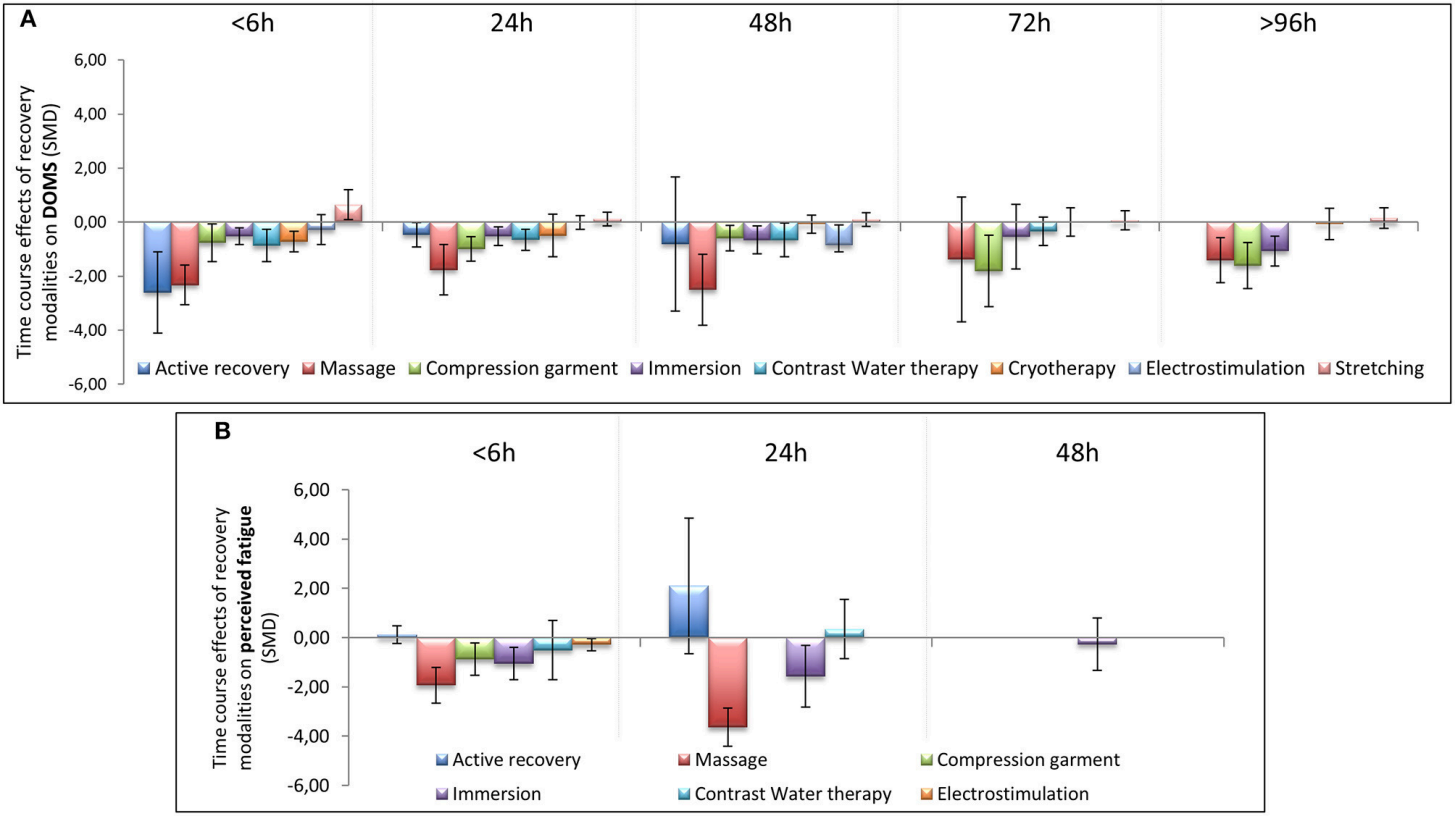
Time course effect of recovery modalities on DOMS **(A)** and perceived fatigue **(B)**. The results are presented as SMD ± IC (SMD, standardized mean differences; IC, interval of confidence).

**Figure 6:**
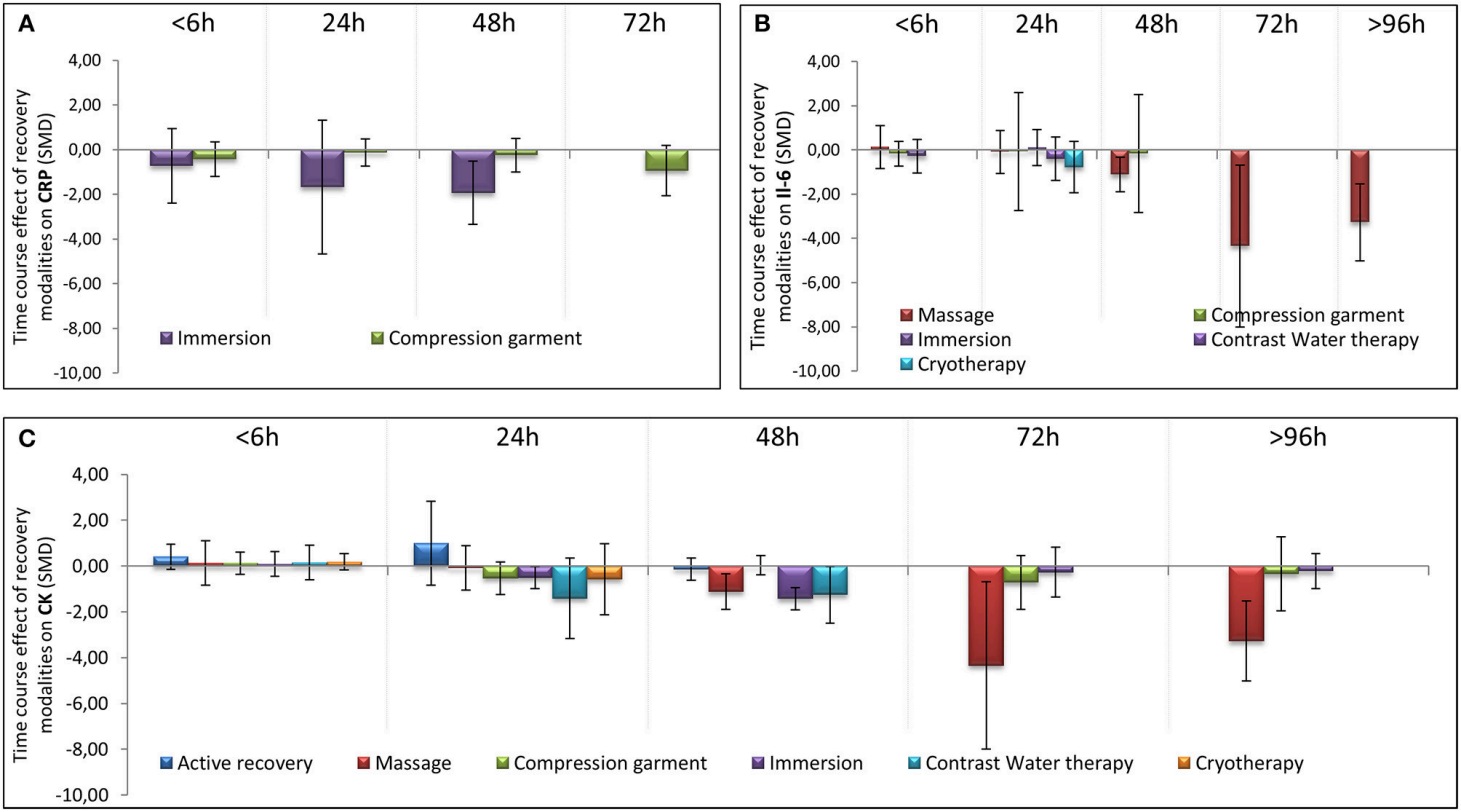
Time course effect of recovery modalities on CRP **(A)**, IL-6 **(B)**, and CK **(C)**. The results are presented as SMD ± IC (SMD, standardized mean differences; IC, interval of confidence).

In terms of population characteristics, the only difference we found was a greater effect of a recovery intervention on the magnitude of DOMS (but not on the magnitude of perceived fatigue) in males than in females (−2.07 < Hedge's g for the difference < −0.43, Z = 4.44, *p* < 0.01). We found no effect of exercise characteristics on the efficiency of a recovery intervention on DOMS, perceived fatigue, markers of inflammation or muscle damage. In contrast, the time course of recovery was modulated by the characteristics of the techniques. As shown in Table 1, active recovery, massage, compression garments, immersion, contrast water therapy and cryotherapy induced a small to large decrease (−2.26 < g < −0.40) in the magnitude of DOMS, while there was no change associated with the other methods. However, this adaptation was not necessarily associated with a similar magnitude decrease in perceived fatigue. In fact, we found a large decrease in this variable (−2.55 < g < −0.88) following the use of massage, compression garments and immersion, while the other methods had no significant effects. The time course effects of the recovery modalities on DOMS and perceived fatigue are presented in Figure 5. In terms of inflammation and muscle damage markers, we observed an overall moderate decrease in creatine kinase [SMD (95% CI) = −0.37 (−0.58 to −0.16), I^2^ = 40.15%] and overall small decreases in interleukin-6 [SMD (95% CI) = −0.36 (−0.60 to −0.12), I^2^ = 0%] and C-reactive protein [SMD (95% CI) = −0.38 (−0.59 to −0.14), I^2^ = 39%]. The specific effects of each method on these markers are presented in Figure 4, and the time course is shown in Figure 6.

**Table 1:** Effects of recovery techniques on the kinetics of DOMS and perceived fatigue.

	**Subjects (*n*)**	**Experimental group (*n*)**	**SMD**	**IC**	**I^2^**
**DOMS**	**1188**	**106**	**−0.78**	**−0.94;** −**0.61**	**56.62**
Active recovery	90	8	−0.94	−1.61; −0.28	^*^
Stretching	67	5	0.15	0.00; 0.29	
Massage	158	14	−2.26	−3.05; −1.47	^*^
Massage + Stretching	N/A				
Compression Garments	160	16	−0.92	−1.34; −0.50	^*^
Electrostimulation	94	8	−0.28	−0.59; 0.03	
Immersion	379	34	−0.47	−0.77; −0.18	^*^
Contrast water therapy	144	12	−0.40	−0.73; −0.07	^*^
Cryotherapy/ cryostimulation	72	6	−0.53	−1.04; −0.03	^*^
Hyperbaric therapy	24	3	0.55	−0.12; 1.22	
**Perceived Fatigue**	**266**	**27**	**−1.40**	**−1.92;** −**0.89**	**32.65**
Active recovery	33	4	0.64	−0.43; 1.70	
Stretching	30	1	−0.21	−1.04; 0.62	
Massage	64	7	−2.55	−3.49; −1.62	^*^
Massage + stretching	9	1	−4.34	−7.20; −1.47	^*^
Compression Garments	28	3	−0.88	−1.34; −0.50	^*^
Electrostimulation	11	1	−0.28	−0.59; 0.03	
Immersion	75	8	−1.16	−1.94; −0.39	^*^
Contrast water therapy	16	2	−0.04	−0.86; 0.79	
Cryotherapy/ cryostimulation	NA				
Hyperbaric therapy	NA				

SMD, standardized mean differences; IC, interval of confidence; NA, not available;

* *significant; − indicates a decrease and + indicates an increase in DOMS and perceived fatigue after the recovery strategy*.

### Effect of the immersion characteristics on DOMS, perceived fatigue, inflammation, and muscle damage

As shown in Table 1, we found a positive effect of immersion and contrast water therapy on the magnitude of DOMS [SMD (95% CI) = −0.47 (−0.77 to −0.18), I^2^ = 50.55% and −0.40 (−0.73 to −0.07), I^2^ = 17.07%, respectively]. In terms of perceived fatigue, we observed a positive effect following immersion [SMD (95% CI) = −1.16 (−1.94 to −0.39), I^2^ = 41.13%], while there was no change after contrast water therapy [SMD (95% CI) = −0.04 (−0.86 to 0.79), I^2^ = 0.00%]. The effect of each immersion procedure (i.e., immersion level and water temperature) on DOMS and perceived fatigue is presented in Table 2. These specifics effects of immersion on inflammation and muscle damage markers are presented in Figure 3. With the exception of immersion in warm water (>36°), all the procedures were associated with similar improvements.

**Table 2:** Effects of the characteristics of immersion on the kinetics of DOMS and perceived fatigue.

	**Subjects (*n*)**	**Experimental group (*n*)**	**SMD**	**IC**	**I^2^**
**DOMS**					
Whole Body	129	12	−0.50	−0.96; −0.04	^*^
Leg	271	25	−0.49	−0.78; −0.20	^*^
≤15°C	234	23	−0.62	−0.93; −0.32	^*^
≤15°C and ≥ 38°C	116	10	−0.40	−0.73; −0.07	^*^
≥ 36°	30	3	0.53	−0.44; 1.51	
**PERCEIVED FATIGUE**					
Whole Body	11	1	0.00	−1.03; 1.03	
Leg	80	9	−1.40	−2.22; −0.58	^*^
≤15°C	75	8	−1.16	−1.94; −0.39	^*^
≤15°C and ≥ 38°C	16	2	−0.04	−0.86; 0.79	

SMD, standardized mean differences; IC, interval of confidence; NA, not available;

* *significant; − indicates a decrease and + indicates an increase in DOMS and perceived fatigue after the recovery strategy*.

## Discussion

In this meta-analysis, we compared the impacts of a single session of different kinds of recovery techniques after physical exercise on DOMS, perceived fatigue, inflammatory [interleukin-6 (IL-6), C-reactive protein (CRP)], and muscle damage markers [creatine kinase (CK)]. The different recovery techniques we investigated were the following: active recovery, stretching, massage, massage combined with stretching, the use of compression garments, electrostimulation, immersion, contrast water therapy, cryotherapy/cryostimulation, and hyperbaric therapy/stimulation. Massage was found to be the most powerful procedure that induced significant benefits in DOMS and perceived fatigue, regardless of the subjects (athletes, sedentary subjects). Additionally, both the use of compression garments and immersion induced a significant positive impact on the same variables but with a less pronounced effect. Active recovery, contrast water therapy and cryotherapy had a positive impact only on DOMS, whereas massage combined with stretching also induced benefits in perceived fatigue. The other techniques did not have a significant impact on DOMS or fatigue but had some effects on circulating CK, IL-6, and CRP concentrations in the blood.

### Massage

Massage is a very traditional way to improve recovery after physical exercise both in sports and rehabilitation contexts, and we found a positive effect of this practice. DOMS is induced by muscle damage, and massage may increase muscle blood flow and reduce muscle oedema (Weerapong et al., 2005; Bakar et al., 2015). A 20–30 min massage that is performed immediately following or up to 2 h after exercise has been shown to effectively reduce DOMS for 24 h after exercise (Torres et al., 2012). Moreover, our results are in accordance with a recent meta-analysis that reported that massage decreases DOMS for 72 h after exercise (Guo et al., 2017). We even found a significant impact up to 96 h after exercise.

In elites athletes (ultra-marathon runners), massage generated a significant improvement in lowering perceived pain (Visconti et al., 2015). Such an improvement might impact perceived fatigue, and our meta-analysis revealed that massage was the most effective technique to reduce perceived fatigue. Following intense cycle pedaling, Ogai et al. (2008) clearly found that perceived fatigue was more effectively reduced by massage than by passive rest without massage. A reduction in circulating cortisol (Field et al., 2005) and an increase in the concentration of beta-endorphins (Kaada and Torsteinb, 1989) have been proposed to explain the reduction of perceived fatigue following massage. Indeed, a 16% rise in the concentration of beta-endorphins in the plasma has been reported following a 30-min massage after exercise (Kaada and Torsteinb, 1989).

Creatine kinase, interleukin-6 and C-reactive protein have often been evaluated in studies concerning DOMS and fatigue, as muscle damage and inflammation are involved (Connolly et al., 2003). In the present meta-analysis, we observed that massage was the most effective recovery technique for reducing the concentrations of circulating CK and IL-6 in the blood after exercise. A decrease in the CK concentration in the blood might reflect a reduction in muscle damage and also indicate a faster recovery after exercise (Bishop et al., 2008). Torres et al. (2012) showed that massage was effective in alleviating the symptoms of exercise-induced muscle damage and was associated with a decrease in plasma CK activity after eccentric exercise (Zainuddin et al., 2005). By increasing blood and lymph flow, massage treatment blunts the CK response due to enhanced CK efflux from damaged tissues and increased CK clearance from the blood (Smith et al., 1994). Furthermore, it has been assumed that massage improves the flushing of neutrophils from the injured area thereby preventing fiber necrosis and CK efflux (Zainuddin et al., 2005). Interestingly, a recent report showed a beneficial effect of massage in decreasing the CK level at 48 h and 72 h in male bodybuilders after intense exercise (Kargarfard et al., 2016), and this effect was confirmed in a recent meta-analysis (Guo et al., 2017).

Massage may also modulate immune compounds when applied after exercise, and these compounds may have a direct impact on fatigue and signs of exercise-induced muscle damage (Tejero-Fernández et al., 2015). In an elegant study in which subjects underwent a series a biopsies of the vastus lateralis following a period of rest after exercise combined with massage on one leg and passive recovery on the other, massage therapy was shown to attenuate inflammatory signaling and IL-6 expression in muscles after exercise-induced muscle damage (Crane et al., 2012). Almost no data are available concerning the effect of massage on CRP concentrations in the blood. Combining stretching with massage has recently been studied (Delextrat et al., 2014). A significant reduction in fatigue was shown. However, discrepancies in the results were found in males and females, with a faster and shorter reduction in fatigue in females (Delextrat et al., 2014).

### Compression garments

Similar to massage, both the use of compression garments and cold water immersion induced a significant and positive impact on DOMS and perceived fatigue but had a less pronounced effect. It has been previously reported in a narrative review (MacRae et al., 2011) and meta-analysis (Hill et al., 2014; Marqués-Jiménez et al., 2016) that the use of compressive garments after damaging exercise affected DOMS. Moreover, recent works confirmed these findings and showed that the effects were still significant at 96 h after exercise (Marqués-Jiménez et al., 2016). Our present study confirmed the significant impact of compression garments on DOMS at 96 h after exercise. We also found a significant decrease in perceived fatigue when compression garments were used. It has been shown that wearing a whole-body compression garment over a 24-h period after intense heavy resistance training significantly reduces perceived fatigue (Kraemer et al., 2010). The beneficial effect of compression garments on DOMS and perceived fatigue might be explained by a possible reduction in the space available for swelling and oedema due to the compression applied to the limb, smaller changes in osmotic pressure that might diminish fluid diffusion in the interstitial space and better venous return (Kraemer et al., 2001).

It has been suggested that using a compression garment may also reduce exercise-induced muscle damage and inflammation (Kraemer et al., 2004). However, in our meta-analysis, we did not observe any significant changes in CK, IL-6, or CRP concentrations after the use of compression garments following exercise. This result is in contrast with a former meta-analysis that found that the use of compression garments was effective for reducing the CK concentration (Hedges' g = 0.44, 95% CI 0.17–0.70) (Hill et al., 2014). In the present meta-analysis, the inclusion criteria were quite stringent, and this certainly influenced the number and the quality of the selected studies. Additionally, in the scientific literature concerning CK concentration and compression garments, there is a very wide range of study protocols. Some discrepancies in the findings exist and may nevertheless be explained by the different recovery period lengths, the lengths of the applied compression (Rimaud et al., 2010), the amount of pressure applied and the place (upper limbs, lower limbs, and even on whole-body) where the compression is applied (Beliard et al., 2015; Brown et al., 2017; Hill et al., 2017). Additionally, individual variability in the sensitivity to blood flow changes may also be of importance (Bishop et al., 2008; Leeder et al., 2012). Similar remarks can be made for the absence of changes in IL-6 and CRP concentrations. Moreover, it previously been expressed that in the majority of the published works concerning the use of compression garments, the exercise protocols were not intense enough to induce a sufficient degree of muscle damage (Pruscino et al., 2013; Bieuzen et al., 2014b).

### Immersion

The effect of cold water immersion (CWI) on DOMS and perceived fatigue was significant, but the effect size was small in terms of DOMS. A previous meta-analysis indicated that CWI induced a significant effect that was still observable at 96 h after exercise when compared with passive recovery (Bleakley et al., 2012). In the same publication, a lower rating of perceived fatigue was observed after CWI (Bleakley et al., 2012). The improvement in overall fatigue through the use of CWI has been reported in several circumstances after training and competition (e.g., soccer tournaments or basketball matches) (Rowsell et al., 2011; Delextrat et al., 2014). Another meta-analysis showed a positive effect (SMD = 0.52) of CWI on lowering DOMS after strenuous exercise in trained athletes and in untrained subjects or those who exercised recreationally (Leeder et al., 2012). An exposure of 11–15°C over 11–15 min was considered to be the optimal circumstance to obtain a positive impact of CWI after exercise to reduce DOMS (Machado et al., 2016). In our meta-analysis, we were able to detect significant differences depending on the water temperature. We observed that only immersion in water with a temperature lower than 15°C had a positive impact on inflammation. Depending on the type of exercise, the duration of immersion, the level of immersion and the water temperature, the outcomes were sometimes at variance.

Several mechanisms have been presented to explain the benefits of CWI on recovery (Ihsan et al., 2016). A common explanation of the impact of CWI on DOMS and fatigue is a reduction in exercise-induced inflammation and muscle damage. We found a significant positive effect of CWI on the CK concentration in the blood after intensive exercise, but the effect size was small. A moderate decrease of circulating CK has previously been reported with the use of CWI after exercise (Leeder et al., 2012; Sanchez-Ureña et al., 2015). Both the level of immersion and the cold temperature of the water may reduce the formation of oedema and pain sensation after exhaustive physical exercise (Wilcock et al., 2006a,b). Hydrostatic pressure may facilitate the transport of fluids from the muscle to the blood and therefore eliminate metabolites (Wilcock et al., 2006a,b; Leeder et al., 2012). Vasoconstriction due to cold temperature may also reduce fluid diffusion into the interstitial space (Eston and Peters, 1999) and locally diminish the inflammatory reaction (Coté et al., 1988), which in turn may reduce the feeling of pain (Smith, 1991). Cold alone has also a direct analgesic impact (Leppäluoto et al., 2008). However, some other authors did not detect any changes in the CK concentration after the use of CWI following exhaustive physical exercise (Bleakley et al., 2012; Hohenauer et al., 2015). No significant effect was observed in our meta-analysis concerning changes in IL-6 and CRP concentrations in the blood. A similar observation has already been presented (Halson et al., 2008; Hohenauer et al., 2015).

### Contrast water therapy

Contrast water therapy (CWT) is often used for recovery purposes and consists of bathing alternately in warm and cold water. We found that CWT had a significant impact on DOMS (though the effect size was small) but not on perceived fatigue. A systematic review has previously expressed the significant impact of using this technique to improve recovery (Hing et al., 2008). A more recent work also showed the effectiveness of this technique after various forms of exhaustive and damaging exercises (Bieuzen et al., 2013). CWT was also able to significantly reduce the perception of pain at 24, 48, and 72 h post-eccentric exercise (Vaile et al., 2008). Alternating therapy with cold and warm water immersion induces successive peripheral vasoconstriction and vasodilation (Bieuzen et al., 2013) and may reduce the formation of oedema after exercise, influence inflammatory pathways and decrease the feeling of pain (Higgins and Kaminski, 1998). Additionally, in the present meta-analysis, we found that CWT reduced CK concentrations in the blood, indicating reduced muscle damage.

### Active recovery

Active recovery (AR) had a similar effect to CWT on DOMS (but with a larger effect size) with no impact on perceived fatigue. The effect of AR after exhaustive exercise on DOMS has been known for more than 30 years (Armstrong, 1984). However, the impact of AR is only significant during a short period after exercise (Zainuddin et al., 2006). In our meta-analysis, we were not able to show a superior influence of AR compared with the other recovery techniques we studied. Similarly, when scrutinizing the scientific literature, no extra beneficial effect of AR was observed after high-intensity eccentric exercise when compared with the benefits obtained from massage and electrostimulation (Weber et al., 1994). We were not able to detect any influence of AR on CK, IL-6, and CRP concentrations in the blood. However, the impact of AR on the CK concentration may depend on the duration of treatment. For instance, after a rugby contest, 1 h of low-intensity aquatic exercise had no impact on the circulating CK concentration (Suzuki et al., 2004), whereas 7 min of low-intensity exercise enhanced CK clearance (Gill et al., 2006). The significant effect of AR may be explained through enhanced blood flow in muscle tissue, which facilitates the removal of metabolic waste, and may contribute to a reduction in muscle lesions and pain (Cheung et al., 2003).

### Cryotherapy

Cryotherapy/cryostimulation was effective in reducing DOMS after exercise but had a rather low effect size. The impact may, however, be at variance. This is probably due to the large amount of heterogeneity among the cryostimulation methods that were used (e.g., exposure in a cold chamber or in a cryo-cabin where the head was not exposed; the timing of the exposure either immediately following or 24 h after exercise; the temperature that ranged from −30 to −195°C; and the number of cryostimulation treatments that were used after exercise) and the exhaustive exercises chosen to induce DOMS and fatigue. With a cryotherapy/cryostimulation exposure organized after a physical exercise, Fonda et al. (Fonda and Sarabon, 2013) found a positive effect of whole body cryotherapy (WBC) on DOMS after an exposure of 3 min exposure at −140 and −195°C. However, Guilhem et al. (2013) and Vieira et al. (2015) did not find such an effect. In their meta-analysis, Costello et al. (2015) did not have sufficient evidence to recommend WBC for preventing muscle soreness. However, our results were in accordance with the conclusions presented in a recent review by Lombardi et al. (2017), in which the use of WBC was associated with an improvement in muscle fatigue, pain, and well-being. However, the present meta-analysis revealed that the effect on DOMS exists shortly after exposure, as we found a positive effect <6 h after exercise. However, this effect is not present after 24 h or later. Thus, cryotherapy performed 24 h after the end of exercise is ineffective in alleviating DOMS. Additionally, it seems that a single cryostimulation treatment does not influence CK and CRP concentrations in the blood after exercise. However, the situation may change if a series of cryostimulation treatments are applied. It has previously been reported that the regular use of cryostimulation after training may lead to lowering concentrations of both CK (by 30–40%) (Wozniak et al., 2007; Banfi et al., 2009) and CRP in the blood (Pournot et al., 2011). In our meta-analysis, a single cryostimulation treatment after exercise induced a significant decrease in IL-6 concentration in the blood.

### Stretching/electrostimulation

In the present work, we did not find any significant influence of stretching or electrostimulation on DOMS and fatigue. For some time stretching has not been recommended after exercise (Herbert and Gabriel, 2002; Herbert et al., 2011), as it might even lead to an increase in DOMS (Smith et al., 1993). The overall SMD in our study confirmed that stretching had no positive impact on DOMS. Moreover, our results at <6 h after exercise indicated that stretching might even produce DOMS, as initially reported by Smith et al. (1993). In terms of electrostimulation, some studies showed positive effects on DOMS (Denegar and Huff, 1988; Denegar and Perrin, 1992), and some found no effect (Craig et al., 1996; Butterfield et al., 1997). However, the protocols used for electrostimulation were different, which may explain, in part, the discrepancy of the outcomes (Babault et al., 2011).

## Limitations

Despite the scientific interest raised by this meta-analysis, the methodology is not free of limitations. Both publication and language restriction bias may have inflated estimates of the effect. Therefore, care was taken to control for these sources of bias. Two of the databases used in the literature search (*Kinpubs* and *Sport-Discus*) included theses and dissertations, thereby allowing access to “gray literature.” Some other limitations specific to the topic of this meta-analysis probably limited the thoroughness of the analyses. For example, the efficiency of a particular recovery technique depends on the type of fatigue induced by previous exercise. Although we coded the characteristics of the training load, it was not possible to address its interaction with the magnitude of the effect, and it might constitute the cornerstone of the global efficiency of a particular recovery strategy. Additionally, the quality of the papers was evaluated according to the PEDro and Van Tulder scales, which are widely used for assessing the methodological quality of clinical tests. A completely blind procedure involving the participants or assessors was impossible for the recovery modalities that were applied. Thus, the attainable maximum score of these scales was 8. However, the quality heterogeneity (varying from 4 to 8) of the included studies was controlled by statistical analysis. A sensitive analysis was performed to determine whether the quality score of the included studies was of importance. We found that the outcomes of our analyses were similar when the studies of lower quality (quality score in Pedro scale lower than 5) and those of higher quality (quality score in Pedro scale higher than 5) were included. Therefore, no bias in our results stemmed from the quality of the included studies. Additionally, given the practical difficulty of blinding the participants, investigators, and outcome assessors from the recovery modalities, the beneficial effect arising from the placebo effect of recovery techniques could not be eliminated.

Some very recently described recovery techniques, such as laser therapy and vibration platforms, were not included in this work. The main reason was because there were a very low number of studies that fulfilled our inclusion criteria, which did not allow the calculation of an effect size to compare with the other recovery techniques.

Finally, in the present work, our main focus was to evaluate muscle damage, soreness, perceived fatigue and inflammation, since these variables are important in short- and long-term recovery processes. Changes in these variables during the recovery period might yield some insights about peripheral and central recovery mechanisms. Since there can be a mismatch between blood-based measures of inflammation or soreness and the recovery of short-term muscular performance (Minett and Duffield, 2014), performance would also have been an important outcome. However, this factor was not evaluated in this manuscript. Performance is a complex concept, and there are many ways to measure it. In many studies, tests and measures used to assess performance capacity are often not reliable or comparable. Additionally, many studies do not provide performance data. As stipulated (Hausswirth and Le Meur, 2011), recovery can be defined as the return to homeostasis of various physiological systems following metabolic and inflammatory challenges and muscle damage induced by exercise training sessions. When the athlete can meet their previous level of performance, this does not mean that the athlete has fully recovered from the previous session of exercise, particularly if perceived fatigue, muscle damage, DOMS and inflammation persist.

Although these limitations should be accounted for, it is worth noting that this is the first meta-analysis to compare several recovery modalities. Additionally, this meta-analysis may be a widely applicable tool for sports scientists, coaches, and medical staff.

## Conclusion

In conclusion, we were able to identify several recovery techniques that can be used after a single exercise session to induce a reduction in DOMS and/or perceived fatigue. Among them, massage seems to be the most effective for both DOMS and perceived fatigue. Water immersion and the use of compression garments also have a significant positive impact on these variables but with a less pronounced effect. Perceived fatigue can be effectively managed using compression techniques, such as compression garments, massage, or water immersion. Furthermore, the most powerful techniques that provide recovery from inflammation are massage and cold exposure, such as water immersion and cryotherapy. In this meta-analysis only one recovery session was examined. Further research should be conducted in order to obtain a clearer picture of possible recovery techniques that best match athlete performance. It is of importance to know if similar outcomes are obtained when the same recovery technique is used on a regular basis after exercise. Additionally, studies combining several recovery techniques should be undertaken to investigate whether synergetic phenomena occur.

## Author contributions

WD, DT, and OD have realized the research literature. WD and DT have coded the studies. WD, DT, OD, LB and BD have written the manuscript.

### Conflict of interest statement

The authors declare that the research was conducted in the absence of any commercial or financial relationships that could be construed as a potential conflict of interest.
